# Smartphone-Based Muscle Relaxation for Migraine in the Emergency Department: A Randomized Clinical Trial

**DOI:** 10.1001/jamanetworkopen.2025.34221

**Published:** 2025-10-16

**Authors:** Mia T. Minen, Elizabeth K. Seng, Benjamin W. Friedman, Alexis D. George, Kristina M. Fanning, Ryan C. Bostic, Scott W. Powers, Richard B. Lipton

**Affiliations:** 1 Department of Neurology, New York University Langone Health, New York; 2 Ferkauf Graduate School of Psychology, Yeshiva University, New York, New York; 3 Department of Neurology, Albert Einstein College of Medicine, New York, New York; 4 Doctoral Program in Psychology, The Graduate Center, City University of New York, New York; 5 MIST Research and Statistical Consulting, North Carolina; 6 Department of Pediatrics, Cincinnati Children’s Hospital, Cincinnati, Ohio; 7 Department of Population Health, New York University Langone Health, New York

## Abstract

**Importance:**

The emergency department (ED) is a critical point of contact within the health care system and an opportunity to initiate nonpharmacologic migraine treatment.

**Objective:**

To examine whether progressive muscle relaxation (PMR) smartphone-based migraine self-management improved patient-reported outcomes for migraine compared with enhanced usual care.

**Design, Setting, and Participants:**

A randomized clinical trial of the smartphone application RELAXaHEAD with and without PMR. Patients aged 18 to 65 years visiting New York University Langone Health EDs for headache who met migraine criteria and self reported 4 or more migraine days per month were recruited from June 2019 to October 2021 with follow-up at 3 months. Data were analyzed from June 2022 to June 2025.

**Intervention:**

Participants in the intervention group were asked to listen to the app-based PMR for 60 days. Participants in the control group were asked to use the app as a symptom diary.

**Main Outcomes and Measures:**

Primary outcome was change in migraine-related disability (MIDAS). Secondary outcomes were change in migraine-specific quality of life (MSQv2) and monthly headache days (MHDs). Adherence (number of days of diary use, PMR use and total minutes of PMR use over 90-day period) was measured using back-end analytics.

**Results:**

Of the 94 patients (median [IQR] age, 33 [26-45] years; 57 [82.6%] female) randomized (48 control patients and 46 PMR patients), 69 of 94 (73%) had 1 or more follow-up MIDAS scores and constituted the modified intent-to-treat population (35 control patients and 34 PMR patients). The mean (SD) change in MIDAS scores from baseline to 3 months (last observation carried forward [LOCF] used if missing 3-month follow-up data) differed between groups (PMR, 25.09 [29.64] vs control, 6.86 [59.61]; *P* = .01). PMR had nearly double the number of respondents improving by 5 or more MIDAS points (28 of 34 [82.4%] vs 16 of 35 [45.7%] respondents; *P* = .002). There was no difference in MSQv2 domains from baseline to LOCF between PMR and control (mean [SD] role function preventive domain for PMR, 16.9 [24.5] vs control, 11.3 [25.9]); emotional function domain (mean [SD] for PMR, 26.5 [26.9] vs control, 19.8 [38.5]); and role function restrictive domain (mean [SD] for PMR, 18.1 [22.7] vs control, 18.7 [26.8]). Mean (SD) change in MHDs (baseline to 3 months) did not differ between groups (PMR, 2.9 [8.0]; 23 days vs control, −1.6 [6.5]; 25 days).

**Conclusion and Relevance:**

A PMR-based self-management program offered to patients with migraine after ED discharge yielded clinically significant reductions in migraine-related disability.

**Trial Registration:**

ClinicalTrials.gov Identifier: NCT04281030

## Key Points

**Question:**

How does a smartphone-based progressive muscle relaxation (PMR) migraine self-management program impact migraine-related disability, quality of life, and monthly headache days (MHDs) in the emergency department (ED)?

**Findings:**

In this randomized clinical trial that analyzed data from 69 participants, the mean change in Migraine Disability Assessment Scale scores (baseline to 3 months) differed between groups, although no change in migraine-specific quality of life or MHDs occurred. Higher PMR use was associated with greater improvement in migraine-related disability.

**Meaning:**

These findings suggest that for ED patients with migraine, the self-management program had clinically significant improvements in migraine-related disability over 3 months.

## Teaser

This randomized clinical trial evaluates whether progressive muscle relaxation smartphone-based migraine self-management improved patient reported outcomes for migraine compared with enhanced usual care.

## Introduction

Migraine is a primary headache disorder consisting of 4-72 hour long recurrent attacks, with moderate to severe pain intensity and symptoms including nausea, vomiting, photophobia, and/or phonophobia. Migraine affects over 14% of people globally^1^ and is a frequent cause of emergency department (ED) visits worldwide.^2^ Of ED patients diagnosed with migraine, one-third^2^ to three-fifths^3^ had a prior migraine diagnosis. Only 34.2% of ED patients presenting with migraine receive preventive medications.^3^ About one-fifth (21.2%) of patients with 4 or more monthly migraine days and at least 1 prior preventive treatment failure sought care in an ED in the prior year (mean [SD], 3 [5.5] ED visits).^4^

Patients who leave the ED with migraine often have a continuation or a recurrence of symptoms.^5^ Nearly half of patients (46%) discharged from the ED with a migraine diagnosis revisit the ED within 3 months.^5^ Despite ED recidivism, multiple recent reviews on treating headache and migraine in the ED do not mention the possibility of initiating preventive treatment in the ED or through referral from the ED.^3,4,5,6,7^ This is an attractive approach to reducing ED recidivism that could prove cost effective. ED visits provide a teachable moment for introducing migraine management approaches to patients in need of improved management.

Previously, we conducted a single-group study in the ED^8^ using a smartphone application (app) for managing migraine consisting of an electronic headache diary, an evidence-based tool for migraine self-management,^9^ and progressive muscle relaxation (PMR), a grade A, evidence-based treatment for migraine prevention.^9,10,11^ The app has been iteratively developed and evaluated in multiple settings.^8,12,13,14,15,16^ In this novel study, we conducted a randomized clinical trial (RCT) to investigate the impact of an app-based PMR intervention vs enhanced usual care on migraine-related disability (primary), migraine-specific quality of life, and headache frequency (secondary) in the ED setting (across 2 EDs). We hypothesized that those in the PMR group would have significantly larger improvements in migraine-related disability than those in the control group.

## Methods

### Study Design

This was a 2-group parallel-group RCT evaluating the utility of a smartphone app vs enhanced usual care in the ED setting to reduce migraine-related disability over a 3-month period. Participants, investigators, and study statisticians were blinded to treatment allocation. The institutional review board of New York University (NYU) Langone Health approved this study. All participants provided written or electronic informed consent. This manuscript follows the Consolidated Standards of Reporting Trials (CONSORT) reporting guidelines for RCTs. The study protocol and statistical analysis plan are available in Supplement 1.

### Inclusion Criteria

The study design was based on prior work.^8,12,13,14,15,16^ Inclusion criteria were: (1) presenting to the ED for headache; (2) being aged 18 to 65 years; (3) speaking English (at the time, the app was available in English and study resources limited this initial phase 2b study to English speakers); (4) self-reporting 4 or more headache days per month; (5) meeting International Classification of Headache Disorders (ICHD)-3 migraine criteria^17^ based on the American Migraine Prevalence and Prevention screener^18^; (6) owning a smartphone and being willing to participate in a smartphone-based migraine management program, and (7) Migraine Disability Assessment Scale (MIDAS) score greater than 5.^19^

### Recruitment and Randomization

**Figure 1.**
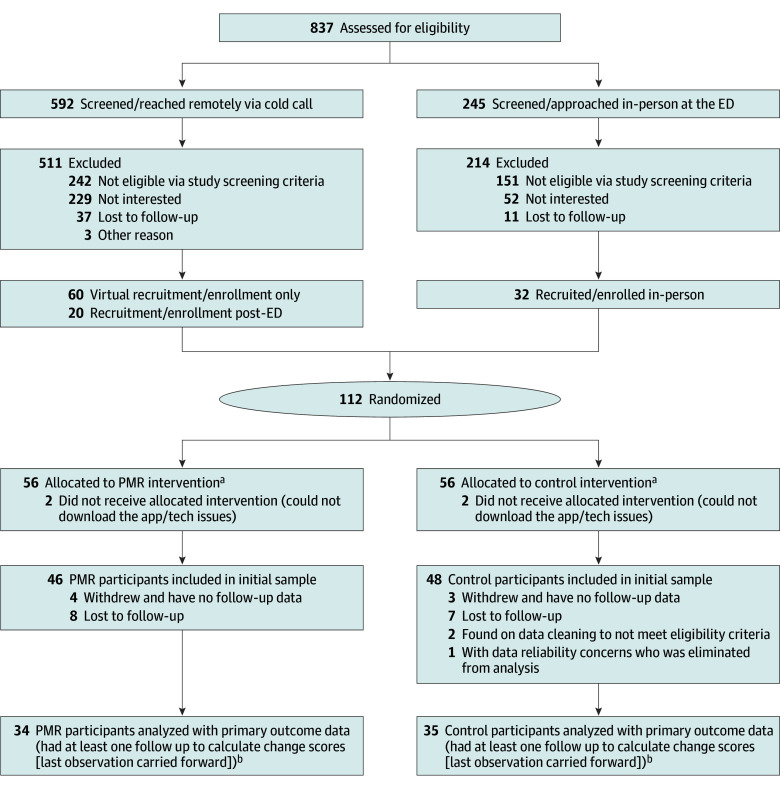
Participant Flow Diagram ED indicates emergency department; MIDAS, Migraine Disability Assessment Scale. ^a^Eighteen participants were deemed ineligible (8 participants had MIDAS <5, and 10 participants did not meet full migraine criteria). ^b^All data entered into the app was included in analyses despite any loss to follow-up; the final number analyzed includes those who formally discontinued answering questionnaires and those lost to follow-up.

Figure 1 illustrates participant flow. Potentially eligible participants were recruited in person in the ED at NYU Langone Health from June 2019 through February 2020. In the summer of 2019, potentially eligible participants were also recruited in person in the ED at NYU Langone Health Brooklyn. Recruitment for the study was paused from March 2020 to June 2020 due to the COVID-19 pandemic. Beginning in July 2020 until October 2021, after the onset of the COVID-19 pandemic, participants were recruited remotely via phone and email based on ED records. Enrollment sessions were approximately 1 hour, in-person or via WebEx. Informed consent was written for in-person enrollments and electronic via REDCap^20^ for virtual enrollments. Participants were randomly allocated in blocks of 4 with a 1:1 randomization ratio before completing the baseline questionnaires. A remotely located statistician generated a randomization list in blocks of 4 using R version 4.1.2 (R Project for Statistical Computing), which was then uploaded into REDCap for the system to allot participants to the 2 groups. Participants were randomized in a 1:1 ratio to the PMR or control groups before completion of the baseline questionnaires.

### Procedures

Participants completed baseline questionnaires via REDCap with the study coordinator (A.D.G.) which included self-report of demographic characteristics and headache history. Participants downloaded and toured the app with assistance from the research coordinator. Participants were informed that the study team could remotely monitor their headache diary (and PMR usage if applicable) throughout the study period. After enrollment, the research coordinator collected all follow-up data questionnaires via phone call and/or email generated by REDCap at 1 week and 1-, 2-, 3-, and 6-month time points (±10 days). Participants were compensated $60 for the initial survey, $1 per day for up to 60 days of data entered, and $2 per day if at least 80% of the days had data entered; $15 for each of the surveys at 1 and 2 months; and $25 for the 3- and 6-month surveys. The maximum total amount was $260.

### Interventions

#### PMR Group

The app is a standardized, evidence-based intervention including a daily symptom-based reporting diary and PMR audio files. The audio files included a 5-minute deep-breathing session, a 6-minute PMR session (brief), a 12.5-minute PMR session (full), and an 8.5-minute muscle scan session (See Table 1 for Protocol). During the review of the app with the research coordinator, participants practiced entering diary data. Those in the PMR group were shown the PMR audio files, were taught the rationale for PMR using a standard script followed by questions and answers, and were instructed to complete the short PMR session. Participants were asked to complete the app-based PMR for 10 to 15 minutes daily (Table 1) for a minimum of 60 days but to aim for daily use during the 90-day treatment period.

**Table 1.** Progressive Muscle Relaxation Protocol^a^

Audio file name	Audio file length
Deep breathing session	5 min
Brief relaxation session	6 min 20 s
Full relaxation session	12 min 30 s
Muscle scan session	8 min 30 s

^a^ Participants were told to use 10 to 15 minutes of PMR each day.

#### Control Group

Participants were asked to use the app as a daily symptom-based reporting diary. This included recording headache days, sleep, medications, medication adverse effects, menstrual cycle, and free text (notes) for optional use. Participants in the control group were not given access to the PMR audio files.

### Measures

In brief, based on our statistician’s scope of work and before conducting the statistical analyses, we defined the primary clinical outcome as migraine-related disability based on MIDAS, a 5-item scale that measures participants’ activity limitations in 3 domains over the past 3 months.^19^ We chose change in MIDAS as the primary outcome to adjust for baseline differences and also reported responder rates using the definition of a clinically meaningful change in MIDAS (reduction of ≥5 points).^21^ Secondary outcomes included migraine-specific quality of life using the MSQv2,^22^ a 14-item measure of quality of life over the past 4 weeks, and headache days measured using the MIDAS question assessing the self-reported number of headache days in the last 90 days at baseline and at 3 months (divided by 3 to calculate monthly headache days [MHDs]). Additional details about the measures (eg, treatment fidelity and Patient-Reported Outcomes Measurement Information System [PROMIS] anxiety and depression scores^23^) can be found in the eMethods in Supplement 1. We also assessed treatment fidelity by assessing the frequency and duration of app use and participant satisfaction with 9 items: 5 items regarding the app (both groups) and 4 items regarding the PMR audio files (PMR group only).

### Statistical Analysis

Detailed descriptions can be found in the eMethods in Supplement 1.^24,25,26,27,28,29^ Briefly, a priori, the sample size was calculated for the primary outcome (change in MIDAS) and the secondary outcome (MHDs) to achieve 80% power and to accommodate a potential dropout rate of 30%. Given the change in the recruitment plan (in-person to remote), we also opted to overenroll participants by about 10%.

Participant and clinical characteristics were described at baseline. Tests for normality were conducted; χ^2^ tests, *t* tests, Mann-Whitney U, and analysis of covariance (ANCOVA) tests were used to evaluate differences between the groups. Independent samples *t* tests evaluated differences in change in MIDAS, change in MSQv2, and change in MHDs between PMR and control groups. In evaluating change in MIDAS, we additionally ran an ANCOVA model that accounted for baseline PROMIS anxiety and depression *t* scores given that both anxiety and depression have been shown to be not just highly comorbid with migraine, but also relevant in affecting migraine prevalence, prognosis, treatment, and clinical outcomes.^30,31^ χ^2^ tests were completed for significant differences in the percentage of PMR and control group improving 5 or more MIDAS points. Logistic regression models for this outcome were adjusted for covariates (number of days of diary use, baseline MIDAS score, and the number of headache days reported in week 1).

To determine if there was a PMR dosing effect, the χ^2^ test for trend evaluated if the percentage of respondents improving 5 or more points on MIDAS differed across 3 groups—control, low PMR use, and high PMR use—with PMR use categorized based on medial split of total minutes of PMR use during the study. ANOVA evaluated differences in change in MSQv2 scores and change in MHDs across usage categories.

Furthermore, as further described and reported in the eMethods in Supplement 2, Generalized linear mixed models were used to examine predictors of diary use throughout the study. The outcome was the number of days participants used the diary per week, with participant ID included as a random effect to account for within-participant correlation. Fixed effects included treatment group, study week, age, and baseline measures: headache pain scale, MIDAS, PROMIS Anxiety and Depression T-scores, MSQv2 subdomains, and the presence of photophobia, phonophobia, and severe headache intensity. All inferential tests were 2-tailed, and *P* values less than .05 were considered significant for all inferential tests and models.

Analyses were conducted in both the modified intention-to-treat (mITT) population, which included participants who initiated treatment and provided at least 1 follow-up, and the completer population, which included participants with baseline and 3-month data. To assess potential attrition bias, noncompleters and completers were compared on baseline demographics, headache features (headache days and intensity), treatment group, recruitment method, and patient reported outcome measures (MIDAS, MSQv2, and PROMIS). All analyses were conducted between June 2022 and May 2025 with SPSS Statistics, version 29.0 (IBM).

## Results

Of the 887 patients who presented to the NYU Langone Health EDs with headache and were prescreened for participation in the study, individuals meeting eligibility criteria (94 participants) were randomly assigned to control (48 participants) or PMR (46 participants) groups (Figure 1). There were no statistically significant differences between those who formally withdrew and those who did not regarding demographic characteristics, treatment group, baseline headaches features (headaches days and intensity), or baseline patient-reported outcomes (MIDAS, MSQv2, and PROMIS).

eTables 1 and 2 in Supplement 2 show the baseline information for all eligible participants, and Table 2 and Table 3 show the baseline information on the mITT sample, those who had data for at least 1 follow-up data collection point (69 participants). Most of the participants (48 of 69 participants [70%]) had complete data at 3 months for MIDAS and MSQv2 domains. For patients missing data at 3 months, we used last observation carried forward (LOCF) to singly impute missing data points (9 participants with 1-month follow-up data and 12 with 2-month follow-up data).

**Table 2.** Baseline Demographics, Overall Medical History, and Relevant Health Care Utilization History

Characteristic	Participants, No, (%)		
	Total (N = 69)	PMR (n = 34)	Control (n = 35)
Age, median (IQR), y^a^	33 (26-45)	31 (25-46)	36 (28-45)
Race			
African American	11 (15.9)	7 (20.6)	4 (11.4)
Asian or Pacific Islander	2 (2.9)	1 (2.9)	1 (2.9)
White	38 (55.1)	17 (50)	21 (60)
Other	18 (26.1)	9 (26.5)	9 (25.7)
Ethnicity			
Hispanic or Latino	21 (30.4)	12 (35.3)	9 (25.7)
Not Hispanic or Latino	48 (69.6)	22 (64.7)	26 (74.3)
Gender			
Male	10 (14.5)	6 (17.6)	4 (11.4)
Female	57 (82.6)	27 (79.4)	30 (85.7)
Other	2 (2.9)	1 (2.9)	1 (2.9)
Medical history			
Psychiatric conditions			
PTSD	10 (14.5)	5 (14.7)	5 (14.3)
Bipolar disorder	6 (8.7)	4 (11.8)	2 (5.7)
Insomnia	3 (4.3)	0 (0)	3 (8.6)
Depression	18 (26.1)	6 (17.6)	12 (34.3)
Anxiety	29 (42)	14 (41.2)	15 (42.9)
Overlapping comorbid pain condition^b^	15 (21.7)	5 (14.7)	10 (28.6)
Prior ED visits (not inclusive of enrollment visit)			
1	30 (43.5)	15 (44.1)	15 (42.9)
2 to 4	18 (26.1)	7 (20.6)	11 (31.4)
≥5	21 (30.4)	12 (35.3)	9 (25.7)
Took medication before ED^c^	56 (83.6)	27 (79.4)	29 (87.9)
Had a primary care physician^d^	58 (85.3)	25 (73.5)	33 (97.1)
Had a physician to treat headache	47 (68.1)	19 (55.9)	28 (80.0)
Family history of migraine	39 (56.5)	20 (58.8)	19 (54.3)

Abbreviations: ED, emergency department; PMR, progressive muscle relaxation; PTSD, posttraumatic stress disorder.

^a^ Missing 2 (1 PMR and 1 no PMR) participants.

^b^ Overlapping pain condition includes temporomandibular disorders, other headache types, chronic fatigue syndrome, endometriosis, chronic low back pain, fibromyalgia, and irritable bowel syndrome.

^c^ Missing 2 no PMR participants.

^d^ Missing 1 no PMR participant.

**Table 3.** Baseline Headache Characteristics

Characteristic	Participants, No. (%)		
	Total (N = 69)	PMR (n = 34)	Control (n = 35)
Headache d/mo, median (IQR)^a^	10 (5-18)	14 (7-20)	9 (5-15)
Headache pain intensity, median (IQR)	7 (6-8)	7 (6-8)	7 (6-9)
MIDAS category			
Moderate (20 or less)	16 (23.2)	9 (26.5)	7 (20)
Severe (>20)	53 (76.8)	25 (73.5)	28 (80)
PROMIS anxiety score, mean (SD)^b^	59.5 (9.7)	59 (10.4)	60.1 (9.1)
PROMIS depression score, mean (SD)	54.5 (8.9)	54.5 (10.1)	54.5 (7.7)
MSQv2_baseline			
Role function restrictive, mean (SD)	41.2 (21.4)	42.5 (23.4)	40 (19.5)
Role function preventive, mean (SD)	54.2 (24.4)	56.9 (26.1)	51.6 (22.5)
Emotional function, mean (SD)	40.7 (26.4)	41 (28.5)	40.4 (24.5)
Prior behavioral treatments tried			
Biofeedback	3 (4.3)	0	3 (8.6)
Progressive muscle relaxation^c^	2 (2.9)	1 (3)	1 (2.9)
Cognitive behavioral therapy	19 (27.5)	8 (23.5)	11 (31.4)
Prior acute medications			
OTC			
Acetaminophen	58 (84.1)	27 (79.4)	31 (88.6)
Ibuprofen	55 (79.7)	26 (76.5)	29 (82.9)
Naproxen (OTC and Rx)	37 (53.6)	19 (55.9)	18 (51.4)
Excedrin	46 (66.7)	24 (70.6)	22 (62.9)
Prescription			
Triptans	33 (47.8)	15 (44.1)	18 (51.4)
Opioids	12 (17.4)	5 (14.7)	7 (20)
Fioricet/fiorinal	9 (13)	4 (11.8)	5 (14.3)
Prior use of migraine preventive medication	29 (42.6)	13 (38.2)	16 (47.1)
Migraine preventive meds			
Calcitonin gene–related peptide antagonist	8 (11.6)	4 (11.8)	4 (11.4)
Onabotulinum toxin^d^	10 (15.2)	2 (6.3)	8 (23.5)
Tricyclic antidepressants	5 (7.2)	4 (11.8)	1 (2.9)
SNRI	3 (4.3)	3 (8.8)	0
Propranolol	3 (4.3)	2 (5.9)	1 (2.9)
Valproic Acid	1 (1.4)	1 (2.9)	0
Lisinopril	1 (1.4)	1 (2.9)	0
Riboflavin	3 (4.3)	2 (5.9)	1 (2.9)
Magnesium	8 (11.6)	5 (14.7)	3 (8.6)
Zonisamide	1 (1.4)	1 (2.9)	0
Other migraine prescriptions	21 (30.4)	7 (20.6)	14 (40)
Current medication(s) effective	48 (69.6)	23 (67.6)	25 (71.4)
Adverse effects from medications	25 (36.2)	10 (29.4)	15 (42.9)

Abbreviations: ED, emergency department; MIDAS, migraine-related disability; MSQv2, migraine-specific quality of life; OTC, over-the-counter; PMR, progressive muscle relaxation; PROMIS, Patient-Reported Outcomes Measurement Information System; Rx, prescription; SNRI, .serotonin-norepinephrine reuptake inhibitor.

^a^ Missing 2 (1 PMR and 1 no PMR) participants.

^b^ Missing 1 no PMR participant.

^c^ Missing 1 PMR participant.

^d^ Missing 3 (2 PMR and 1 no PMR) participants.

As seen in Table 2 and Table 3, the median (IQR) age of the 69 participants in the sample was 33 (26-45) years. The majority of the sample identified as female (57 of 69 participants [82.6%]). Regarding race and ethnicity, respectively, 11 of 69 (15.9%) identified as African American, 18 of 69 (26.1%) as other races, and 38 of 69 (55.1%) as White; and approximately one third of the sample (21 of 69 participants [30.4%]) identified as Hispanic or Latino. Less than half (29 of 69 participants [42.6%]) of participants reported taking daily preventive migraine medication. The study groups were generally balanced for potential confounders including demographic characteristics, headache features (headache days and intensity), rates of prior ED visits, and medication use before their ED visit. The control group was more likely to have a primary care physician (33 of 35 participants [97.1%] vs 25 of 34 [73.5%]; *P* = .01) and to have a physician treating their headache (28 of 35 participants [80.0%] vs 19 of 34 [55.9%]; *P* = .04).

Among the baseline characteristics, only 1 statistically significant difference was observed between the 48 participants in the completer and 46 in the noncompleter populations, all those in the noncompleter population (100%) reported baseline photophobia compared with 40 of 48 (83.3%) of those in the completer population (χ^2^_1_ = 8.380; *P* = .004).

### Primary Outcome: MIDAS

Among the 69 participants (35 in control and 34 in PMR) with at least 1 follow-up MIDAS score, the mean (SD) change in MIDAS score from baseline to LOCF (primary end point) was −25.1 (29.6) for the PMR group and 6.9 (59.6) for the control group. In the completer population, mean (SD) change in MIDAS scores were similar (control, 1.28 [59.6] vs PMR, −31.0 [33.9]). Independent *t* tests showed statistically significant differences between the PMR and control groups in both the mITT and completer populations (*t*_67_ = −2.81; *P* = .01 and *t*_46_ = −2.28; *P* = .03, respectively). Results from ANCOVA models adjusting for baseline PROMIS anxiety and depression T-scores were consistent, with significant differences observed in both populations (*F*_1,64_ = 7.76; *P* = .01 and *F*_1,44_ = 5.13; *P* = .03, respectively). Overall, 44 of 69 patients (63.8%) were responders, showing a clinically meaningful decrease of 5 or more MIDAS points (improvement in migraine-related disability). The PMR group had a higher proportion of responders than the control group (PMR, 28 of 34 [82.4%] vs control, 16 of 35 [45.7%]; χ^2^_1_ = 10.02; *P* = .002). Among the completer population the PMR group had a higher proportion of responders, although the difference was no longer statistically significant (PMR, 17 of 23 [73.9%] vs control, 13 of 25 [52.0%]). When adjusting the responder analysis for number of days of diary use, baseline MIDAS score, and week 1 headache-free days using logistic regression, PMR was still associated with higher odds of responding to treatment vs control (odds ratio [OR], 10.63; 95% CI, 2.58-43.5; *P* < .001). Among the completer population, PMR odds of responding was not statistically significant (OR, 2.26; 95% CI, 0.49-10.58; *P* = .30).

### Secondary Outcomes

#### MSQv2

Increases in MSQv2 domain scores from baseline to LOCF were nominally but not significantly greater in the PMR group compared with the control group for the role function preventive (RFP) domain (mean [SD] PMR, 16.9 [24.5] vs control, 11.3 [25.9]]. For the emotional function (EF) domain, mean (SD) improvements were slightly lower in the PMR group (PMR, 19.8 [38.5] vs control, 26.5 [26.9]). Role function restrictive (RFR) domain change scores were nominally similar between groups (mean [SD] PMR, 18.1 [22.7] vs control, 18.7 [26.8]). Similarly, among the completer population, increases in MSQv2 domain scores from baseline to 3-month follow-up were nominally but not significantly greater in the PMR group compared with the control group for the RFP domain (mean [SD] PMR, 17.0 [24.3] vs control, 15.6 [21.7]). Mean (SD) EF domain score increases from baseline to 3-month follow-up were again nominally greater in the PMR group, but the difference did not reach statistical significance (PMR, 18.3 [39.0] vs control, 32.2 [26.3]). Mean (SD) RFR domain change scores were nominally similar between groups (PMR, 19.25 [25.2] vs control, 21.79 [24.1]).

#### Monthly Headache Days (MHDs)

Completer analysis showed that participants with 12-week MHD data across both groups (48 participants; 23 in PMR and 25 in control) reported a mean (SD) change of −2.2 (7.2) MHDs when comparing baseline with 12-week data. There was no significant difference between the change in mean (SD) MHDs in the groups (PMR, −2.9 MHDs [8.0] vs control, −1.6 [6.5] MHDs).

### Dosing

**Figure 2.**
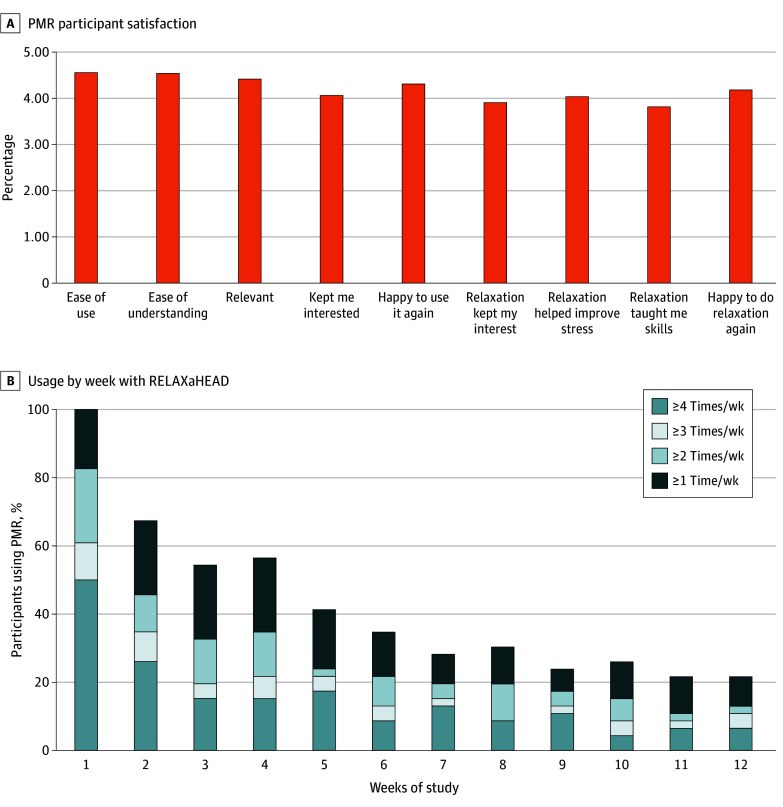
Progressive Muscle Relaxation (PMR) Participant Satisfaction and Usage by Week

Among the 46 participants randomized to PMR (Figure 2), the mean number of days using PMR was nominally but not significantly higher among participants who responded (achieved a 5-point reduction in MIDAS; responders, mean [SD] number of days using PMR over 12 weeks, 18.6 [18.9] vs nonresponders, 12.8 [12.6]). Among the completer population, the mean (SD) number of days using PMR was similar between responders and nonresponders (19.8 [12.9] vs 19.6 [22.9] days). The median (IQR) cumulative total minutes of PMR use was 46.3 (116). Change in MIDAS of 5 or more points was observed in 18 of 20 (90.0%) in the high-use PMR group, 10 of 14 (71.4%) in the low-use PMR group, and 16 of 35 (45.7%) in the control group with a significant linear trend across categories (χ^2^_1_ = 11.03; *P* = .001). Post hoc tests revealed significant differences between the control and high-use diary groups (χ^2^_1_ = 10.38; *P* = .001), but no differences were detected between control and low-use diary groups or low-use and high-use diary groups. Among the completer population, a similar pattern was observed; 9 of 13 participants (69.2%) in the high-use diary group, 8 of 10 (80%) in the low-use diary group, and 13 of 25 (52%) in the control group achieved a 5-point or more improvement in MIDAS. However, these differences were not statistically significant. Post hoc tests comparing the control and low-use diary groups and the control and high-use diary groups did not reach significance.

No statistically significant differences were observed among the PMR use groups (high-use vs low-use vs control) in mITT population for changes in MSQv2 domains. Similarly, in the completer population, there were no significant differences in change scores for MSQv2.

## Discussion

We found that among patients presenting to the ED with migraine, smartphone-based PMR significantly reduced disability as measured by MIDAS scores over a 3-month treatment period. There were statistically significant and clinically meaningful differences in those in the PMR group compared with the control group; those in the PMR group were 10 times more likely to have a 5-point improvement in MIDAS scores compared with the control group. The improvements in MIDAS in the PMR group compared with the control group persisted after adjusting for anxiety and depression. The findings are impressive, especially compared with pharmacologic studies with MIDAS as a primary outcome.^32,33,34,35,36,37,38,39,40,41^

Overall, MSQv.2 scores improved for both the PMR and control groups without statistically significant between-group differences. The study was powered for change in MIDAS and not change in MSQv2, potentially contributing to this effect. Prior psychometric work suggests that MIDAS may be more sensitive to the more severe end of the disability spectrum.^42^ Since patients were recruited from the ED, this study may have enrolled patients with relatively severe migraine-related disability. In addition, over half of the participants had at least 1 prior ED visit. Given the nonsignificant trends, we may require a larger sample to achieve statistically significant differences on MSQv.2.

Few studies have examined the effects of interventions following discharge from the ED. Available studies focus on the role of medications administered in the ED on headache recurrence.^43,44^ An RCT with 50 participants showed that a comprehensive migraine intervention (ie, medications, education, and follow-up with a headache specialist) offered before ED discharge did not improve headache-related disability 1 month after ED discharge.^26^ We found that recruitment after ED discharge requires fewer resources (ie, no need to staff the ED with people trained in the intervention at all hours) than recruitment in the ED.

We tracked minutes of PMR engagement using the app’s back-end analytics. In our previous single-arm ED-based study,^8^ 51 participants completed PMR sessions on a mean (SD) of 13 (19) (range, 0-82) days for the 90-day study period, lasting a median (IQR) of 11 minutes (6.5-17) each. The current study offered more behavioral options; participants spent fewer minutes per session (approximately 6 minutes). Despite the reduction in app engagement, participants had clinically significant improvements in migraine-related disability. The median number of days of diary use were similar between studies (34 days in the single-arm study, and 30 days in the PMR group of this RCT). Both studies revealed a significant improvement in MIDAS scores; in the single-arm study, MIDAS scores significantly decreased by a mean of 38 points per participant (*P* < .001), and in this RCT, MIDAS scores decreased by a median of 25.1 points.

In the single-arm study, more frequent PMR use was associated with higher odds of headache-free days.^8^ In this RCT, participants who used PMR more frequently had greater improvement in migraine-related disability.

The number of days of diary use was higher for the control group compared with the PMR group over the 12-week study period, similar to findings in prior studies.^12,13,14^ This is likely due to the greater time and effort required to interact with the app in the PMR group. Detailed results on diary use can be found in the eAppendix in Supplement 2.

There was little difference in the median number of total minutes of PMR in the first 6 weeks and over the 12-week study period, although the ranges varied. Attrition in PMR use per min, and usage on a weekly basis plateaued around week 6 (Figure 2B). This is similar to the findings in prior RELAXaHEAD studies.^12,16^

### Strengths and Limitations

To our knowledge, this is the first RCT of a migraine preventive treatment for patients with migraine who presented to the ED and one of the few trials assessing a migraine behavioral therapy for migraine presenting to the ED. This is also one of the very few trials reporting treatment fidelity measures of a mobile health behavioral study. We had a racially and ethnically diverse sample; our study population identified as over 30% Hispanic or Latino and 45% as non-White.

This study is limited by the modest sample size and the significant loss to follow-up, which likely worsened due to the COVID-19 pandemic. Another limitation is that we accounted for PMR sessions only if they were recorded with the app’s back-end analytics. We did not capture informal PMR practice without the app. We did not capture changes in preventive medications during the study period. Interviews suggest that some participants learned the PMR techniques and practiced them without the app. Our low rates of formal PMR use may underestimate actual use. The LOCF was used to calculate change scores given the missing diary data. That said, missing data are extremely common in mobile health studies, and our rates align with other such studies.^45,46,47^

## Conclusions

In this randomized clinical trial, smartphone-based relaxation offered to patients who visit the ED with migraine was associated with reduced migraine-related disability. Our results support the ED visit as a feasible method for recruiting patients with migraine for trials and instituting methods to reduce headache-related disability.

## Notes

Supplement 1.
Trial Protocol

Supplement 2. **eMethods.** Supplementary Statistical Analysis **eAppendix.** Supplementary Results **eTable 1.** Baseline Demographics, Overall Medical History, and Relevant Health Care Utilization History **eTable 2. **Baseline Headache Characteristics
eReferences.

Supplement 3.
Data Sharing Statement
