# Supplementary material for: Smartphone-Based Muscle Relaxation for Migraine in the Emergency Department: A Randomized Clinical Trial
Source: JAMA Netw Open. 2025 Oct 16;8(10):e2534221. doi: 10.1001/jamanetworkopen.2025.34221 (PMC12531881; doi:10.1001/jamanetworkopen.2025.34221)
Supplement: Supplement 1. — Trial Protocol [file jamanetwopen-e2534221-s001.pdf]

Supplementary Online Content

Supplement 1. Trial protocol submitted to Institutional Review Board.

Research Protocol Addendum Aim #2

Improving Health Outcomes of Migraine Patients who Present to the Emergency Department

This addendum serves to provide additional information to main protocol (uploaded on Research Navigator). A full bibliography can be found in the main protocol.

Purpose of the Study and Background

P u r p o s e o f t h e S t u d y

Aim 2: Examine the impact smartphone-based PMR on migraine quality of life, frequency, intensity, and disability.

Aim 3: Determine whether markers such as stress, anxiety and mood disorders predict patient response to an ED-initiated PMR therapy. High levels of baseline pain, scores on the Perceived Stress Scale and the PROMIS anxiety and depression measures may predict response to the comprehensive intervention on migraine disability.

B a c k g r o u n d

The ED is a critical point of contact with the health care system for many migraine patients; in current practice, it is a missed opportunity to initiate and establish a comprehensive migraine management paradigm. Behavioral headache treatments (e.g., progressive muscle relaxation (PMR), biofeedback, cognitive-behavioral therapy (CBT)) are effective migraine treatment options that are essentially free of side effects. [31] Behavioral treatments have enduring benefits [32] and may be less costly than pharmacologic interventions. [33] PMR is a standardized, evidence-based behavioral treatment used for migraine since the 1980s. [34,35] **PMR has also been successful as a technique that patients can do independently.** [36,37]

Migraineurs in the ED continue to experience pain and are discharged without adequate treatment plans. Less than 25% of ED migraine patients are pain-free on discharge. [39,40] For most patients, headache returns within 24 hours of discharge. [40] About 40% of patients are not prescribed medication at discharge [40] and most patients are not advised to follow-up with a physician. [40,41] There are no clear guidelines for ED management of migraine. [42] Therefore, a simple, effective comprehensive intervention is needed for patients who present to the ED with migraine.

Migraineurs' response to interventions, including the ability to adhere to medication regimens and behavioral interventions, may also depend on various factors, such as mood, anxiety, and stress. Headache patients with comorbid major depressive disorder were less likely to adhere to preventive medications. [43] Behavioral therapy decreases depressive symptoms and improves adherence to pharmacologic treatment in other disorders. [44] Thus, research is also needed to examine what markers might be associated with response to an ED intervention for migraine. [45]

Smartphone applications (Apps) improve collection of migraine outcome data. Apps with electronic headache diaries are a reliable method for data collection preferred over paper headache diaries by patients because the Apps are more discreet in the work place. [46] Apps for collecting patient reported outcome data have become possible due to widespread use of smartphones, [47,48] and result in fewer secondary data errors, [49] less administrative burden, [50,51] high participant acceptance, [51] and potential cost savings. [52] Additionally, Apps allow the use of reminders and timely follow-up of non-compliant participants via real time investigator data

monitoring capabilities. Most migraineurs who present for care are between ages 18–50, and >80% of Americans in this age group have smartphones. [53] Furthermore, an increasing number of ED patients have smartphones.[54-56] In this study, all subjects will use an MCIT approved App developed by IRODY to track their headaches. Subjects in the intervention group also will use the App to access PMR therapy and record PMR frequency in real time.

Study Design

In Aim 2, the PMR behavioral smartphone app will be used by enrolled migraine subjects in the ED.

Subjects will be recruited from the New York University Medical Center (NYUMC) Perelman Emergency Care Center, a large urban ED in New York City with ~65,000 adult subjects/year. The ED has a research volunteer program with research volunteers available for screening subjects for studies in the ED. In a 2-month window (4/22–6/22/14), there were 190 primary diagnoses with a chief complaint of “headache” who presented to the NYU ED, projecting 1040 primary headache diagnoses annually. Thus, with a recruitment period of 2 years (Years 2, 3) in Aim 2, there will be time to enroll the proposed 90 subjects.

For Aim 3, we will use data acquired in Aim 2 to explore predictors of subject response to the PMR intervention. For continuous and categorical outcomes, the effects of the treatment with baseline pain, psychiatric comorbidity, and stress levels on subject outcomes will be examined using linear regression models or generalized linear models, respectively. The multiplicative interaction terms will be created by multiplying the treatment indicator variable with baseline risk factors, and then included in the model. Given the exploratory nature of this aim, we will first examine the baseline factors of interest one-by-one and their significance, and consider multiple factors and their interactions simultaneously. Variable selection and model checking techniques will be considered to derive parsimonious and appropriate models.

Characteristics of the Research Population

Number of Subjects

We had approval for initial beta testing and then for 90 subjects to be recruited. To date (April 2019), we have recruited 22 subjects in the ED and 44 subjects post ED discharge for Aim 1. Now that we have worked to optimize the app and study design, we are requesting that we be able to recruit up to 90 subjects for Aim 2.

This is a pilot study.

Research volunteers in the ED will evaluate which patients are eligible to participate based on the inclusion and exclusion criteria stated below. During the time of covid 19, the research data associate will determine inclusion and exclusion eligibility remotely as described below.

Gender of Subjects

Both genders will be included.

Age of Subjects

18-65

We are excluding patients above the age of 65 because we want the same type of subjects to trial the app as those who will be eligible for the future study with the app.

Racial and Ethnic Origin

No enrollment restrictions.

I n c l u s i o n C r i t e r i a

age 18–65; Meets migraine criteria based on Information in Study Manual or based on Headache expert opinion if RA has a question and Headache expert, Dr. Minen, determines that migraine criteria are still met, 4+headache days a month.

E x c l u s i o n C r i t e r i a

Patients who have had Cognitive Behavioral Therapy, Biofeedback or other Relaxation Therapy in the past year; Cognitive deficit or other physical problem with the potential to interfere with behavioral therapy; Alcohol or other substance abuse as determined by self-report or prior documentation in the medical record; Unable or unwilling to follow a treatment program that relies on written and audio recorded materials; Not having a smartphone.

V u l n e r a b l e S u b j e c t s

N/A

Methods & Procedures

Methods & Procedures

Aim 1 has been completed. We are proceeding with the Methods & Procedures for Aim 2:

Aim 2: **Examine the impact smartphone-based PMR on migraine quality of life, frequency, intensity, and disability.** The primary outcome is to assess whether use of PMR in the ED improves migraine quality of life (MSQv2) at 3 months post ED-discharge (or post enrollment date if recruited post ED discharge). All subjects (N=90) will complete a migraine quality of life assessment and track their headache frequency and intensity using our APP. The smartphone app will be used to monitor adherence.

The non-traditional Research Volunteer (RV) or Research Assistant (RA) will obtain subject consent for the study.

In the light of COVID-19 and increased participant preference for remote visits, we will also recruit subjects via telephone and electronic consent will be obtained via REDCap. Following this, the initial enrollment visit will be scheduled and completed via Webex. The followup data points will continue to be collected via followup calls or emails as approved previously.

The NYU Headache Research Program has been recognized for its solid training of pre-health students in research methods. In fact, it received the first ever City College of New York S Jay Levy Fellowship for Future Leaders Distinguished Internship Host Award (2017-2018).

We carefully select from a group of CUNY students who are part of the highly coveted S Jay Levy Fellowship. These students are pre-selected by CUNY for their professionalism and top grades and then the students undergo a very selective interview process by Dr. Minen. We also take high achieving Barnard students who are pre-health who have high grades and many of whom are part of the Barnard Summer Research Institute. Dr. Minen has been a mentor as part of this program for several years, and the students in this program are supposed to learn research methods from start to finish, culminating in a poster presentation at a minimum. We have attached a copy of the training curriculum to this protocol based on the training that was done last year. In brief, the students take the online CITI training and pre-read selected articles prior to the on-site training. Then, there are four full days of one-site training led by Dr. Minen the first week followed by an additional week of shadowing the already trained research team. Dr. Minen then observes each student the second week with a participant to ensure treatment fidelity.

All study team members will have undergone a two week training intensive to understand the screening

process, recruitment process, informed consent process and enrollment process (as described throughout this IRB). In brief, research volunteers will be trained during an intense two week training on study procedures

including an emphasis on how to screen, recruit, consent, and enroll the participants. This is a no risk/very low risk study unlike other interventional studies. It is similar to many of the hundreds of commercial apps available on the market that people use every day. Thus, it is appropriate for trained research volunteers to consent these participants just as they would be able to do so for survey type studies. The study team members will be carefully observed during the initial consent process to ensure that they are doing it correctly.

While explaining the study, potential subjects will be assured that they will receive all of the medication for their acute headache that they would otherwise receive. Once consented, subjects will be randomized to PMR therapy or MUC. Participants will be told that either group is being tested as an enhancement to the care they typically get in the ED for headache. At the end, they will be told that they will be informed which group they were in. The RV or RA will then collect subjects' health histories (biologic variables) and baseline data in REDCap on an iPad, download the app onto subjects' smartphones, and conduct the sessions (see below). There will be fidelity monitoring.

We will be requesting the Epic Research Team for a report of the following data for patients who visited the ED for headache from 10/22/2017 to 02/18/2019:

- gender
- race
- pcp or neurology care provider
- zip codes - or region they live in
- did they have a neurologist before visiting ED
- did they have diagnosis of migraine prior to visiting the ED
- had they seen a neurologist prior to visiting the ED
- did they attend a follow up appointment
- did they have a Rx for triptans prior to visiting the ED
- did they have a Rx for opiods prior to visiting the ED

PMR: The RV or RA will explain the rationale for PMR and the APP will be uploaded onto the subjects' smartphones. The subject will perform PMR in the ED and discuss the optimal time and place to practice PMR at home.
Subjects will also enter their headache log daily on the APP. The APP will track the amount of time playing the PMR. Subjects will be asked to do the following:

Intervention:
Week 1: 5 min deep breathing at least 5/7 days of the week
Week 2: 5 min PMR session at least 5/7 days of the week
Week 3: 15 min pmr preferred at least 5/7 days of the week
Week 4: pmr at least 4 days a week (optional body scan)
Week 5: pmr at least 3 days a week (optional body scan)
Weeks 6-8: use when it is most helpful (falling asleep, HA coming on, stress)

The study intervention will be 8 weeks in duration but the full study length will be 6 months. There will be a baseline assessment (T0), a f/u call or email at one week (T1), a 30 day phone call or email assessment (T2), a 60 day phone call/email (T3), and a 90 day phone call/email (T4) (to examine persistence of effect after a month). The last follow-up will be at 6 months to examine persistence of effect 4 months after the intervention ends. As we learned previously, we are adding the option for email as some subjects prefer email.

MUC: To match the time spent with the designated study team member, subjects will be given a general education session consisting of basic migraine information, such as evidence-based ways to treat headaches: treat early, limit acute medications < 2–3 days/week, and call the primary care physician (PCP) if abortive medications are used more frequently. They will be shown a website: https://miachen26.wixsite.com/relaxahead (to be renamed) The study team member will load the app onto the subject's smartphone but the PMR component will be blocked on the version of the app that the MUC subject receives.

Discharge Plans and Post-Discharge Plans for All Subjects: All subjects will receive written educational material about migraine. This is the standard information about migraine that is printed from Epic when patients are discharged from the ED with migraine. Subjects will be asked to follow-up with a PCP upon discharge from the ED and will be offered an appointment for this follow-up visit unless they have an appointment scheduled. Subjects without a PCP will be referred to a PCP. There will be emphasis on ensuring that the subjects understand the discharge information, which will include the diagnosis and both verbal and written instructions. Letters will be sent to PCPs informing them of the study and stating that they are to treat the subjects as they see fit.

All subjects will receive follow-up phone calls or emails (from REDCap or send safe emails) at 7 days and 1, 2, 3, and 6 months after discharge. Follow-up will be assured by testing the subject's phone number in front of the subject and asking for alternative contact information. Prior studies have shown that data can be successfully obtained from subjects at 24 hours, and 1 and 3 months-post ED discharge (over 90% follow-up rates). During the calls, the RV or RA will reiterate the importance of adherence with the APP. During the 1, 2, 3 and 6 month calls or emails (from REDCap or send safe emails), they will be asked to complete the REDCap follow-up questionnaires (already part of the IRB submission with the added modification of the MSQv2). They may also receive phone calls or SendSafe emails or texts up to every 3 days if the smartphone app data indicates that they are not completing the headache diary. If benefits do not persist, future studies may add additional interventions. Throughout the study, subjects will be asked to record electronically any new and/or discontinued medications and interactions with the medical community.

The choice of calls, texts or emails came from prior subjects who participated in IRB approved study focus groups. The study team has permission from MCIT to send the following text messages (and an emailing indicating such approval is part of the submission process):

1. Please remember to use the app.
2. Hi, The RELAXaHEAD team is trying to reach you. Please call xxx-yyy-zzz [study phone number]?
3. You are due for your follow-up call/email. Please be sure to call us at xx-yyy-zzzz or respond to the email. -The RELAXaHEAD team.
4. Remember-relaxation is beneficial for your health.
5. The best way to add relaxation into your routine is to pair it with an activity you do each day. Let us know if you want to discuss how to improve your adherence with the RELAXaHEAD app.
6. Research has suggested that the more one is able to practice relaxation, the better one's health. Log into the RELAXaHEAD app when able.

All subjects will be given a $60 gift card incentive for participating, and will receive an additional $1 per day they do the intervention to compensate them for study-related time and data usage costs. If they complete the intervention more than 80% of the total 60 days of the study, then the daily compensation will be doubled. In addition, they will receive $15 gift cards for the one month and two month f/u emails/calls and $25 gift cards for the 3 month and 6 month f/u email or phone assessments. Thus, based on the prior adherence data, the max total projected amount that participants will receive is $200 but if they get the bonus, they could get up to $260.

This payment is on par with other migraine smartphone studies throughout the country.

Fidelity monitoring will be conducted as in Aim 1.

Data Analysis and Data Monitoring

Analysis Plan

Sample Size: The sample size was calculated for the primary outcome (ΔMIDAS) and the secondary outcome (number of headaches/30 days). A study of medication plus behavioral therapy for migraine as compared with medication alone in the outpatient setting showed that the distribution of MIDAS at baseline or 3 months was right-skewed, but the ΔMIDAS was symmetric and approximately normally distributed. Considering ΔMIDAS as the primary outcome can reduce data skewedness and improve study power. Based on the study, the standard deviation (SD) of ΔMIDAS ranges from 5 to 7. With the conservative estimate of SD=7, group sample sizes of 31 and 31 achieve 80% power to detect a clinically significant difference of 5 between the groups, assuming

group standard deviations of 5 and a significance level of 0.05 using a two-sided two-sample t-test. The emergency medicine studies had over 90% follow-up at 3 months and the electronic headache diary studies' follow-up was between 76%–80%. To accommodate potential dropout rate as high as 30%, a total of 90 subjects will be recruited.

For the secondary outcome of number of headaches in 30 days, group sample sizes of 31 and 31 achieve 80% power to detect a difference of 1.42 assuming an SD of 2 in both groups with a significance level of 0.05 using a two-sided two-sample t-test. Potential non-normal distributions will be considered for the count data of the number of headaches and analyzed using nonparametric tests. A simulation-based sample size calculation was performed assuming Poisson distributions. The sample size of 31 per group has >80% power to detect the difference of 1.4 using a two-sided Mann-Whitney test at significance level 0.05 assuming a Poisson distribution with mean 2 in the control group.

Aim 2 Data Collection and analyses:

PHI will be maintained in a separate log maintained securely as described below under data security.

Symptoms Diary

On the smartphone application platform developed by IRODY (used by the NYUMC epilepsy group for ongoing IRB approved studies), subjects will be trained to keep records of headache occurrence, side effects, compliance and medicine changes on their mobile device. If a subject fails to consistently record their data, they will receive a call/text/email from the RV or RA to ensure compliance with data maintenance. Information prompted by the device will include:

1. Headaches (number, type, and dates)
2. Abortive and preventive migraine drug treatment
3. Concomitant medications
4. Episodes of non-compliance
5. Changes made to migraine medication treatment plan, if any, and reasons for the change
6. Adverse effects, including severity, seriousness, unexpectedness as part of pharmacovigilance
7. Mood and anxiety levels, stress and sleep
8. Healthcare utilization

The symptoms diary may be accessed via the mobile application only. The app also includes the following questionnaires: PROMIS for depression and anxiety, the Perceived Stress Scale, and the Insomnia Sleep Index.

The study team will not share the symptoms diary information with the subject's treating physician. However, subjects can elect to share their data with their treating physician independently.

In addition to the application, REDCap, a HIPAA Compliant research database system, generated emails, and telephone calls by the blinded RAs with data entered into REDCap will be used to collect the MSQv2 and the MIDAS. The PHI of the subjects will only be stored in REDCap. IRODY does not receive PHI in the app or log in process.

Quality Control: The Clinical Data Monitor will download the data from the Diary Cloud Server. From this download, standard reports including missing days or entries as well as identification of any safety concerns will be created from the downloaded database and examined and reviewed by the Clinical Data Monitor.

Measurements: Our primary outcome, the Migraine Quality of Life (MSQv2) is a validated quality of life measure. In addition, we will check the Migraine Disability Assessment Scale (MIDAS), measures disability over the past 3 months. MIDAS scores were shown to have excellent test-retest reliability, high internal consistency and high external validity using a variety of measures including diary based measures. MIDAS scores are also associated with physician judgements about the severity of headache and the need for treatment. A decrease of 3 points in the MIDAS score corresponds to a one day reduction in headache related disability per month, a clinically meaningful difference. In the Headache Management Trial, persons with headache were randomized to coordinated, guideline-based care vs. usual care. The study was designed to detect a 5-point difference in MIDAS decline between treatment arms and found a highly clinically significant 7 point decline. We will examine the change in disability scores (∆MIDAS) defined as the difference between MIDAS scores at 3 months and at baseline.

Data Storage and Confidentiality

Each subject will be assigned a unique alphanumeric identifier for use with the app. No PHI is being given to IRODY. The alphanumeric identifiers will be unrelated to the identity of the study subjects. All clinical data/information will be entered into the RedCap database. Only the PI and the RVs/RAs will have access to the linking database.

The consent forms will be kept in a separate locked file cabinet in the Tisch ED. They will be transferred periodically to a locked cabinet at 222 East 41st Street, 14th floor. If someone were to gain illegal access to the locked filing cabinet with study data, they would have no way to link this data to any identifying information.

The research team will perform all data analyses. Data will be stored electronically in a secure research database and will be available for the research team.

Research subjects will be assured that participation or non-participation in this investigation will not influence in any way the patient's treatment by his or her healthcare providers, and will not influence any future interactions he or she may have with NYU Langone.

The application produced by the company IRODY has been used by other researchers in the department of neurology at NYUMC. The symptoms diary on the mobile device used by subjects will be secured to comply with data privacy and other regulatory requirements. Data security is maintained by having a secure data protocol, which encrypts the information being sent from the mobile device to the cloud server and vice versa. The server is protected by a firewall. Daily backups will be performed. The app will not hold any personally identifying detail and will be linked only to the Subject ID. The app does not collect any personal information from the mobile device. As stated above, the Data Analyst will have access to the symptoms diary data, as well as all subjects' names and phone numbers, in order to contact any participants who forget or are unable to enter their information in a timely manner. The Data Analyst will access the symptoms diary data only for the purpose of ensuring data completion and will not disclose any subjects' PHI.

The audio-recording will be recorded via a portable audio-recording device. The subject's name will not be on the device-only the identifier. The audio files will be uploaded to an online transcription service. The audio files will be maintained on the HIPAA compliant NYULMC server in case there ever needs to be re-review of the files.

Risk/Benefit Assessment

Risk

Risks from PMR: There is minimal harm in doing PMR therapy. PMR may cause some pain in previously injured sites. It may also cause some flooding of memories. If this occurs, subjects will be directed to turn their thoughts elsewhere. If this method is ineffective, subjects will be told that they can stop performing the relaxation.

Potential Breaches of Confidentiality: There is a small risk of breach of confidentiality for all Aims. Protections against this are described.

Protection Against Risks

As stated above, subjects will be warned of the side effects.

Potential Benefits to the Subjects

Subjects may benefit by having their migraine pain decrease in severity and frequency with the PMR.

Investigator's Qualifications & Experience
Please see attached.

Subject Identification, Recruitment and Consent/Assent

Method of Subject Identification and Recruitment
Research volunteers/assistants will identify subjects in the ED for the study.

Process of Consent
Research volunteers/assistants will obtain consent as described in the process above.

Subject Capacity
Only subjects with capacity will be asked to participate in the study.

Subject/Representative Comprehension
Subjects will be asked to explain that they understand that they will complete surveys and may be offered a smartphone application with a relaxation exercise to help relax them to try to decrease the pain.

Subjects will be asked to explain that they understand that they will be asked to do a relaxation exercise and that they will be asked to record their headache characteristics, mood, anxiety and stress levels on the smartphone app as well as the number of hours that they slept.

Debriefing Procedures

N/A

Data Safety Monitoring Plan
The PI will be responsible for data safety monitoring. This is a minimal risk device. Minimal to no risk is expected for participants in the study. Only the PI and co-investigators will have access to the data. No personal/identifying information will be kept on an insecure site. No personal or identifiable information will be stored within the app. We will report all unanticipated events within a 24- to 48-hour time window or as soon as possible. There are no predetermined criteria for action.

Consent Forms

A consent form for each subject will be obtained. Please see attached addendum consent.

Documentation of Consent
The documentation will be kept in a locked storage unit in the NYUMC ED initially and will periodically be moved to a locked storage unit in Dr. Minen's office on the 14th floor of the ACC.

Payment for Participation
All subjects will be given a $60 incentive for participating, and will receive an additional $1 per day they do the intervention to compensate them for study-related time and data usage costs. If they complete the intervention more than 80% of the total 60 days of the study, then the daily compensation will be doubled. In addition, they will receive $15 for the one month and two month f/u emails/calls and $25 for the 3 month and 6 month f/u email or phone assessments. Thus, based on the prior adherence data, the max total projected amount that participants will receive is $200 but if they get the bonus, they could get up to $260.

This is on par with other smartphone migraine studies done at other institutions.

Cost to Subjects:

Subjects will have to pay for routine follow-up visits to the primary care physician and/or neurologist. These visits are not for research purposes and are typically offered to migraine patients in the ED setting anyway.
