# Supplementary material for: Smartphone-Based Muscle Relaxation for Migraine in the Emergency Department: A Randomized Clinical Trial
Source: JAMA Netw Open. 2025 Oct 16;8(10):e2534221. doi: 10.1001/jamanetworkopen.2025.34221 (PMC12531881; doi:10.1001/jamanetworkopen.2025.34221)
Supplement: Supplement 2. — eMethods. Supplementary Statistical Analysis eAppendix. Supplementary Results eTable 1. Baseline Demographics, Overall Medical History, and Relevant Health Care Utilization History eTable 2. Baseline Headache Characteristics eReferences. [file jamanetwopen-e2534221-s002.pdf]

Supplemental Online Content

Minen MT, Seng EK, Friedman BW, et al. Smartphone-based muscle relaxation for migraine in the emergency department: a randomized clinical trial. *JAMA Netw Open*. 2025;8(9):e2534221. doi:10.1001/jamanetworkopen.2025.34221

eMethods. Supplementary Statistical Analysis

eAppendix. Supplementary Results

eTable 1. Baseline Demographics, Overall Medical History, and Relevant Health Care Utilization History

eTable 2. Baseline Headache Characteristics

eReferences.

This supplemental material has been provided by the authors to give readers additional information about their work.

© 2025 Minen MT et al. *JAMA Network Open*.

eMethods. Supplementary Statistical Analysis

Missing Data

The analyses reported below included two distinct populations: the modified intention-to-treat (mITT) population and the completer population. The mITT population comprised all participants who initiated treatment and provided at least one follow-up measurement. For this group, missing data for the primary endpoint (ΔMIDAS) and secondary endpoints (ΔMSQv2 subscales) were handled using the Last Observation Carried Forward (LOCF) method to ensure all participants contributed to the analysis. To assess the robustness of findings, these key analyses were also ran on the completer population which included only participants who completed the 3-month follow-up assessment. No imputation was performed for this population; only participants with complete baseline and 3-month follow-up data were included in the analysis. Monthly Headache Days (MHD) analyses were limited to the completer population.

Primary Clinical Outcome

MIDAS, a 5-item scale that measures participants' activity limitations in 3 domains over the past 3 months.[1] Items assess missed work or school, housework and social/leisure days, and days where productivity was reduced by $\geq$50% due to migraine.[1] Last observation carried forward (LOCF) was used to impute missing longitudinal MIDAS responses for mITT population. We chose change in MIDAS (ΔMIDAS) as the primary outcome as it is typically normally distributed. Change in Migraine-Related Disability (ΔMIDAS) was calculated by subtracting baseline MIDAS score from 3-month score for completers and LOCF for mITT; negative values indicate a decrease in disability. We also reported clinically significant change in MIDAS (reduction of $\geq$5 points).[2]

Other Clinical Outcomes

The MSQv2 is a 14-item measure of quality of life over the past 4 weeks. It has 3 domains: Role Function Restrictive (RFR), Role Function Preventive (RFP), and Emotional Function (EF).[3] Scores range from 0 to 100, with high scores indicating better quality of life.[3] Migraine-Specific Quality of Life (MSQv2) change scores were calculated for each domain by subtracting participants' baseline MSQv2 score from 3-month score for completers and LOCF for mITT; positive values indicate increased quality of life.

Headache days were measured using the MIDAS question assessing the self-reported number of headache days in the last 90 days at baseline and at 3 months (divided by 3 to calculate monthly headache days (MHDs)). Change in Monthly Headache Days (MHDs) was created by subtracting baseline MHDs from 12-week MHDs, with negative values indicating reduction in MHDs.

Other Measures

Treatment fidelity was measured by the following: (1) The frequency and duration of RELAXaHEAD app use collected using the app's backend analytics, including: days of diary use (both arms), and number of days and cumulative minutes of PMR (PMR arm only). A median split of cumulative minutes designated a "low PMR use" and a "high PMR use" group. (2) Participant satisfaction with the RELAXaHEAD app was assessed during the first week of the study with 9 items: 5 regarding the app broadly (both arms) and 4 regarding the PMR audio files (PMR arm only). Response range of Strongly Disagree (1) to Strongly Agree (5); these items had been used in prior RELAXaHEAD studies.[8,12-16]

© 2025 Minen MT et al. *JAMA Network Open*.

PROMIS anxiety and depression short-form measures[4] (each 8-items, with responses ranging from 1 [Never] to 5[Always]) assessed psychiatric symptoms at baseline; sums are compared to norms and presented as t-scores, with higher scores indicating higher psychiatric symptoms.

Power Calculation
Based on prior research,[5] the standard deviation (SD) for ∆MIDAS ranges from 5 to 7. With the conservative estimate of SD=7, group sample sizes of 31 and 31 achieve 80% power to detect a clinically significant difference of 5 between the groups, assuming group standard deviations of 5 and a significance level of 0.05 using a two-sided two-sample *t*-test. Given that emergency medicine studies had over 90% follow-up at 3 months[6–8] and electronic headache diary studies' follow-up was between 76%–80%,[9,10] we wanted to accommodate a potential 30% dropout rate. Thus, we planned to recruit 45 patients per group. For the secondary outcome of MHDs, group sample sizes of 31 and 31 achieve 80% power to detect a difference of 1.42, assuming an SD of 2 in both groups with a significance level of 0.05 using a two-sided two-sample *t*-test. A simulation-based sample size calculation was performed, assuming Poisson distributions. The sample size of 31 per group has >80% power to detect the difference of 1.4 using a two-sided Mann-Whitney test at a significance level of 0.05, assuming a Poisson distribution with a mean of 2 in the control group. Ultimately, given the change in the recruitment plan (in-person to remote), we opted to over-enroll participants by about 10%.

Statistical Tests Used
Histograms, qq plots, and Kolmogorov-Smirnov tests evaluated normality of continuous distributions. Nominal variables were described using frequency counts and percents. Chi-square tests evaluated differences for nominal variables. For continuous variables with an approximate normal distribution, mean (SD) was reported and independent group *t*-tests were used to evaluate differences between PMR and control arms. For non-normally distributed continuous variables, median, interquartile range (IQR) was reported and Mann-Whitney U was used to test for differences between PMR and control arms.

Independent samples T-test evaluated differences in ∆MIDAS, ∆MSQv2, and MHDs between PMR and control arms; ANCOVA adjusted for covariates (baseline PROMIS anxiety and depression). Chi-square tested for significant differences in the percentage of PMR and control arm improving ≥5 MIDAS points; logistic regression adjusted for covariates (number of days of diary use, baseline MIDAS score, and the number of headache days reported in week one).

Generalized linear mixed models assessed predictors of diary use during the study. The number of days of diary use per week was the outcome, with subject ID as the random effect. Fixed effects included treatment group, week, age, baseline scores for headache pain scale, MIDAS, PROMIS anxiety and depression, MSQv2 subdomains, and baseline presence of photophobia, phonophobia, and severe headache intensity. An independent covariance structure was chosen based on goodness of fit statistics.

© 2025 Minen MT et al. *JAMA Network Open*.

eAppendix. Supplementary Results

Fidelity Measures
Figure 2a presents the satisfaction scores for various aspects of the intervention on a 5-point scale. For items applicable to both the PMR and Control arms, participants rated “Ease of use” and “Ease of understanding” the highest, with mean scores of 4.56 and 4.54, respectively. These results suggest high overall usability and clarity of the intervention. For items specific to the PMR arm, such as “Relaxation taught me skills” (mean score: 3.82) and “Relaxation helped improve...” (mean score: 4.03), responses were only available from participants in the PMR arm (n = 34).

Diary Use
Among the mITT population (n=69; PMR=34, Control=35) the median number of days of diary use in the 12-week period across both arms was 60 (IQR 60.8), with the PMR arm using the diary on significantly fewer days than the control arm [PMR, 37 (IQR 56.3); Control, 74.5 (IQR 46), p=0.013]. Similar results were found among completers (n=48; PMR=23, Control=25) [PMR, 73 (IQR 35.0); Control, 84.0 (IQR 3), p=0.005]. A generalized linear mixed model was used to analyze predictors of diary use, with diary use treated as a binary variable (used or not used in each trial) and modeled using a Binomial distribution with a logic link function. The model included baseline demographics, headache characteristics, and headache burden measures as predictors. The model found study arm assignment was a statistically significant (B=-1.069, SE =0.42, p=0.010) predictor of diary use, with similar results found among completers (B=-1.532, SE =0.62, p=0.014). Participant use of PMR by week is shown in Figure 2b.

© 2025 Minen MT et al. *JAMA Network Open*.

eTable 1. Baseline Demographics, Overall Medical History, and Relevant Health Care Utilization History

	Statistics	PMR	No PMR	p-value	Total
**Age**	Median (N) [IQR]	31 (46) [25-43]	36 (48) [28-46]	0.179	33.5 (94) [26-45]
**Race**				0.928	
African American	n/N (%)	10/46 (21.7)	10/48 (20.8)		20/94 (21.3)
Asian or Pacific Islander	n/N (%)	1/46 (2.2)	1/48 (2.1)		2/94 (2.1)
White/Caucasian	n/N (%)	23/46 (50.0)	27/48 (56.3)		50/94 (53.2)
Other	n/N (%)	12/46 (26.1)	10/48 (20.8)		22/94 (23.4)
**Ethnicity**				0.131	
Hispanic or Latino	n/N (%)	16/46 (34.8)	10/48 (20.8)		26/94 (27.7)
**Gender**				0.895	
Male	n/N (%)	9/46 (19.6)	8/48 (16.7)		17/94 (18.1)
Female	n/N (%)	36/46 (78.3)	39/48 (81.3)		75/94 (79.8)
Other	n/N (%)	1/46 (2.2)	1/48 (2.1)		2/94 (2.1)
**Medical History**					
**Psychiatric Conditions**					
PTSD	n/N (%)	7/46 (15.2)	6/48 (12.5)	0.771	13/94 (13.8)
Bipolar Disorder	n/N (%)	7/46 (15.2)	2/48 (4.2)	0.088	9/94 (9.6)
Insomnia	n/N (%)	1/46 (2.2)	3/48 (6.3)	0.617	4/94 (4.3)
Depression	n/N (%)	12/46 (26.1)	16/48 (33.3)	0.503	28/94 (29.8)
Anxiety	n/N (%)	19/46 (41.3)	20/48 (41.7)	1.000	39/94 (41.5)
**Overlapping Pain Condition***	n/N (%)	6/46 (13.0)	15/48 (31.3)	0.034	21/94 (22.3)
**Prior ED Visits**				0.536	
1	n/N (%)	20/46 (43.5)	16/48 (33.3)		36/94 (38.3)
2-4	n/N (%)	13/46 (28.3)	14/48 (29.3)		27/94 (28.7)
5+	n/N (%)	13/46 (28.3)	18/48 (37.5)		31/94 (33.0)
**Took Medication prior to ER**	n/N (%)	35/46 (76.1)	38/46 (82.6)	0.607	73/92 (79.3)
**Has a primary care physician**	n/N (%)	30/46 (65.2)	45/47 (95.7)	<0.001	75/93 (80.6)
**Has a physician to treat HA**	n/N (%)	23/46 (50)	35/48 (72.9)	0.033	58/94 (61.7)
**Family History of Migraine**	n/N (%)	27/46 (58.7)	25/48 (52.1)	0.541	52/94 (55.3)

© 2025 Minen MT et al. *JAMA Network Open*.

eTable 2. Baseline Headache Characteristics

	Statistics	PMR	No PMR	p-value	All
**HA Characteristics**					
**Sound Sensitivity**	n/N (%)	40/46 (87.0)	43/48 (89.6)	.692	83/94 (88.3)
**Light Sensitivity**	n/N (%)	42/46 (91.3)	44/48 (91.7)	1.000	86/94 (91.5)
**HA Days per Month**	Median (N) [IQR]	14 (45) [6-20]	10 (47) [5-16]	0.235	10 (92) [5-17]
**Average HA Pain Intensity**	Median (N) [IQR]	7 (46) [6-8]	7.5 (48) [6-9]	0.258	7 (94) [6-8]
**MIDAS**				0.629	
Moderate (20 or less)	n/N (%)	12/46 (26.1)	10/48 (20.8)		22/94 (23.4)
Severe (>20)	n/N (%)	34/46 (73.9)	38/48 (79.2)		72/94 (76.6)
**PROMIS Anxiety**	(N) Mean ± SD (Min-Max)	(46) 59 ± 10.2 (37.1-77.4)	(47) 59.1 ± 9.1 (37.1-82.8)	0.809	(93) 59 ± 9.6 (37.1-82.8)
**PROMIS Depression**	(N) Mean ± SD (Min-Max)	(46) 54.7 ± 10.4 (37.1-73.7)	(48) 53.8 ± 8.2 (37.1-70.3)	0.739	(94) 54.2 ± 9.3 (37.1-73.7)
**MSQv2_baseline**					
**Role Function Restrictive**	(N) Mean ± SD (Min-Max)	(46) 42 ± 22.8 (5.7-100)	(48) 42 ± 19.5 (2.9-80.0)	0.940	(94) 42 ± 21.1 (2.9-100)
**Role Function Preventive**	(N) Mean ± SD (Min-Max)	(46) 58 ± 24.6 (0-100)	(48) 55 ± 22.0 (0-95.5)	0.501	(94) 56.3 ± 23.2 (0-100)
**Emotional Function**	(N) Mean ± SD (Min-Max)	(46) 41 ± 27.8 (0-100)	(48) 41 ± 26.1 (0-93.3)	0.885	(94) 41 ± 26.8 (0-100)
**Prior Behavioral Treatments Tried**					
Biofeedback	n/N (%)	**0**	4/48 (8.3)	0.117	4/94 (4.3)
Progressive Muscle Relaxation	n/N (%)	1/45 (2.2)	1/47 (2.1)	1.000	2/92 (2.2)
Cognitive Behavioral Therapy	n/N (%)	14/46 (30.4)	14/48 (29.2)	1.000	28/94 (29.8)
**Prior Acute Medications**					
**OTC**					
Acetaminophen	n/N (%)	37/46 (80.4)	41/48 (85.4)	0.590	78/94 (83)
Ibuprofen	n/N (%)	37/46 (80.4)	37/48 (77.1)	0.803	74/94 (78.7)
Naproxen (OTC & Rx)	n/N (%)	28/46 (60.9)	23/48 (47.9)	0.222	51/94 (54.3)
Excedrin	n/N (%)	33/46 (71.7)	30/48 (62.5)	0.385	63/94 (67)
**Prescription**					
Triptans	n/N (%)	18/46 (39.1)	25/48 (52.1)	0.222	43/94 (45.7)
Opioids	n/N (%)	6/46 (13)	9/48 (18.8)	0.576	15/94 (16)
Fioricet/Fiorinal	n/N (%)	5/46 (10.9)	7/48 (14.6)	0.759	12/94 (12.8)
**Prior Use of Migraine Preventive Medication**	n/N (%)	16/46 (34.8)	23/47 (48.9)	0.209	39/93 (41.9)
**Migraine Preventive Meds**					
Calcitonin gene related peptide antagonist	n/N (%)	4/46 (8.7)	4/48 (8.3)	1.000	8/94 (8.5)
Onabotulinum Toxin	n/N (%)	2/43 (4.7)	10/47 (21.3)	0.029	12/90 (13.3)
Tricyclic Antidepressants	n/N (%)	4/46 (8.7)	2/48 (4.2)	0.430	6/94 (6.4)
SNRI	n/N (%)	3/46 (6.5)	0	0.113	3/94 (3.2)
Propranolol	n/N (%)	2/46 (4.3)	1/48 (2.1)	0.613	3/94 (3.2)
Valproic Acid	n/N (%)	1/46 (2.2)	0	0.489	1/94 (1.1)
Lisinopril	n/N (%)	1/46 (2.2)	0	0.489	1/94 (1.1)
Riboflavin	n/N (%)	2/46 (4.3)	1/48 (2.1)	0.613	3/94 (3.2)
Magnesium	n/N (%)	6/46 (13)	3/48 (6.3)	0.311	9/94 (9.6)

© 2025 Minen MT et al. *JAMA Network Open*.

eTable 2. Baseline Headache Characteristics

	Statistics	PMR	No PMR	p-value	All
Zonisamide	n/N (%)	1/46 (2.2)	0	0.489	1/94 (1.1)
Other Migraine Prescriptions	n/N (%)	10/46 (21.7)	21/48 (43.8)	0.029	31/94 (33)
**Current medication(s) effective**	n/N (%)	32/46 (69.6)	35/48 (72.9)	0.821	67/94 (71.3)
**Side Effects from Medications**	n/N (%)	15/46 (32.6)	20/48 (41.7)	0.400	35/94 (37.2)

© 2025 Minen MT et al. *JAMA Network Open*.

eReferences

1. Stewart WF, Lipton RB, Dowson AJ, Sawyer J. Development and testing of the Migraine Disability Assessment (MIDAS) Questionnaire to assess headache-related disability. *Neurology*. 2001;56(6 Suppl 1):S20-S28. doi:10.1212/WNL.56.suppl_1.S20

2. Lipton RB, Desai P, Sapra S. How much change in headache-related disability is clinically meaningful? Estimating minimally important difference (MID) or change in MIDAS using data from the AMPP study. *Headache*. 2017;57(Abstract PF52):113. doi:10.1111/head.13102

3. Speck RM, Shalhoub H, Wyrwich KW, et al. Psychometric Validation of the Role Function Restrictive Domain of the Migraine Specific Quality-of-Life Questionnaire Version 2.1 Electronic Patient-Reported Outcome in Patients With Episodic and Chronic Migraine. *Headache*. 2019;59(5):756-774. doi:10.1111/head.13497

4. Schalet BD, Pilkonis PA, Yu L, et al. Clinical Validity of PROMIS® Depression, Anxiety, and Anger across Diverse Clinical Samples. *J Clin Epidemiol*. 2016;73:119-127. doi:10.1016/j.jclinepi.2015.08.036

5. Matchar DB, Harpole L, Samsa GP, et al. The headache management trial: a randomized study of coordinated care. *Headache*. 2008;48(9):1294-1310. doi:10.1111/j.1526-4610.2007.01148.x

6. Friedman BW, Hochberg ML, Esses D, et al. Recurrence of primary headache disorders after emergency department discharge: frequency and predictors of poor pain and functional outcomes. *Ann Emerg Med*. 2008;52(6):696-704. doi:10.1016/j.annemergmed.2008.01.334

7. Friedman BW, Solorzano C, Norton J, et al. A Randomized Controlled Trial of a Comprehensive Migraine Intervention Prior to Discharge From an Emergency Department. *Acad Emerg Med*. 2012;19(10):1151-1157. doi:10.1111/j.1553-2712.2012.01458.x

8. Friedman BW, Gensler S, Yoon A, et al. Predicting three-month functional outcomes after an ED visit for acute low back pain. *Am J Emerg Med*. 2017;35(2):299-305. doi:10.1016/j.ajem.2016.11.014

9. GIFFIN NJ, RUGGIERO L, LIPTON RB, et al. Premonitory symptoms in migraine: An electronic diary study. *Neurology*. 2003;60(6):935-940. doi:10.1212/01.WNL.0000052998.58526.A9

10. Moloney MF, Aycock DM, Cotsonis GA, Myerburg S, Farino C, Lentz M. An Internet-Based Migraine Headache Diary: Issues in Internet-Based Research. *Headache*. 2009;49(5):673-686. doi:10.1111/j.1526-4610.2009.01399.x

© 2025 Minen MT et al. *JAMA Network Open*.
