# Supplementary material for: Smartphone-Based Muscle Relaxation for Migraine in the Emergency Department: A Randomized Clinical Trial
Source: JAMA Netw Open. 2025 Oct 16;8(10):e2534221. doi: 10.1001/jamanetworkopen.2025.34221 (PMC12531881; doi:10.1001/jamanetworkopen.2025.34221)
Supplement: Supplement 3. — Data Sharing Statement [file jamanetwopen-e2534221-s003.pdf]

Data Sharing Statement

Minen. Smartphone-Based Muscle Relaxation for Migraine in the Emergency Department. *JAMA Netw Open*. Published September 29, 2025. doi:10.1001/jamanetworkopen.2025.34221

Data
Additional Information: ClinicalTrials.gov Identifier: NCT04281030
Data available: Yes
Data types: Deidentified participant data
How to access data: minenmd@gmail.com
When available: beginning date: 01-01-2026

Supporting Documents
Document types: None

Additional Information
Who can access the data: Researchers whose proposed use of the data has been approved
Types of analyses: For a specified purpose, data will be provided per NYU Langone Health and NIH policies
Mechanisms of data availability: After approval of a proposal and with a signed data access agreement
